# The first case report of septic abortion resulting from β-lactamase-negative ampicillin-resistant non-typeable *Haemophilus influenzae* infection

**DOI:** 10.1099/jmmcr.0.005123

**Published:** 2017-10-12

**Authors:** Hiroaki Baba, Risako Kakuta, Hasumi Tomita, Minako Miyazoe, Masatoshi Saito, Chihiro Oe, Noriomi Ishibashi, Misa Sogi, Kengo Oshima, Tetsuji Aoyagi, Yoshiaki Gu, Makiko Yoshida, Koichi Tokuda, Shiro Endo, Hisakazu Yano, Mitsuo Kaku

**Affiliations:** ^1^​ Department of Infection Control and Laboratory Diagnostics, Internal Medicine, Tohoku University Graduate School of Medicine, Miyagi, Japan; ^2^​ Department of Otolaryngology, Tohoku Medical and Pharmaceutical University Hospital, Miyagi, Japan; ^3^​ Department of Gynecology and Obstetrics, Tohoku University Graduate School of Medicine, Miyagi, Japan; ^4^​ Department of General Internal Medicine, Taiyo-kai Social Welfare Awachiiki Iryo Center, Chiba, Japan; ^5^​ AMR Clinical Reference Center, National Center for Global Health and Medicine Hospital, Tokyo, Japan; ^6^​ Department of Infection Control, International University of Health and Welfare Shioya Hospital, Tochigi, Japan; ^7^​ Department of Microbiology and Infectious Diseases, Nara Medical University, Nara, Japan

**Keywords:** sepsis, septic abortion, *Haemophilus influenzae*, β-lactamase-negative ampicillin-resistant (BLNAR), cefotaxime

## Abstract

**Introduction.** This is the first case report of septic abortion due to β-lactamase-negative ampicillin-resistant (BLNAR) non-typeable *Haemophilus influenzae* infection. In Japan, BLNAR *H. influenzae* is widespread and has become a clinical concern, especially in paediatrics and otolaryngology, but *H. influenzae* has not been previously recognized as a causative agent of obstetric or gynaecological infection.

**Case presentation.** A 31-year-old pregnant woman presented at 17 weeks and 6 days of gestation with a high fever; she was admitted with a diagnosis of threatened premature delivery. Despite tocolytic treatment, she aborted spontaneously 2 h after admission and then entered septic shock. BLNAR *H. influenzae* was detected in both blood and vaginal cultures. Her condition gradually improved after several days of treatment with cefotaxime, and she was ultimately discharged without sequelae or complaints.

**Conclusion.** Although penicillin with a β-lactamase inhibitor is currently recommended for the treatment of septic abortion, this combination will probably lead to treatment failure in the case of BLNAR *H. influenzae* infection. As this study reveals, *H. influenzae* can cause septic abortion; hence, future efforts should be undertaken to detect and therapeutically target this pathogen during pregnancy.

## Abbreviations

BLNAR, β-lactamase-negative ampicillin-resistant; Hib, H. influenzae serotype b; MLST, multilocus sequence typing; NTHi, non-typeable H. influenza; PBP3, penicillin-binding protein 3; ST, sequence type.

## Introduction

*Haemophilus influenzae* is a Gram-negative, pleomorphic, facultatively anaerobic coccobacillus that can cause various human diseases, ranging from mild to severe. *H. influenzae* is categorized into six serotypes (a–f) based on capsular polysaccharide composition or is unencapsulated (non-typeable *H. influenzae*: NTHi). Among these, *H. influenzae* serotype b (Hib) is the most virulent strain and can cause life-threatening invasive diseases [1]. The introduction of routine Hib vaccination has resulted in a dramatic reduction in the incidence of invasive Hib disease in many countries, including Japan [2, 3]. However, invasive non-Hib diseases, such as those caused by NTHi and serotypes e and f, are increasing in these regions [4, 5]. In addition, the high prevalence of the β-lactamase-negative ampicillin-resistant (BLNAR) NTHi strain has emerged as a clinical concern, particularly in Japan [6–8].

Sepsis during pregnancy remains an important cause of maternal death [9]. Although septic abortion is now rare in developed countries, the majority of deaths after spontaneous abortion are caused by bacterial infection [10]. Sepsis in pregnancy and septic abortion are mainly caused by group A *Streptococcus*, *Escherichia coli*, and anaerobes such as *Peptostreptococcus* species and *Bacteroides* species [9, 10]. Here, we report a case of septic abortion in Japan resulting from BLNAR NTHi infection for the first time.

## Case report

A 31-year-old previously healthy woman, gravida 2 para 1, presented to her obstetrician at 17 weeks and 6 days of gestation with a high fever accompanied by rigors. Prior to presentation, she showed symptoms of an upper respiratory tract infection, including a mild cough (for 9 days), and light vaginal bleeding with lower abdominal pain (for 4 days). After undergoing a gynaecological examination, she was admitted with a diagnosis of threatened premature delivery. Despite tocolytic treatment, she aborted spontaneously 2 h after admission and then entered septic shock, at which point she was transferred to the Obstetric Department of Tohoku University Hospital for further treatment.

At this point, the patient appeared systematically unwell but was fully aware. The abdomen was soft, not sore to the touch, and was mildly distended. Her vital signs were as follows: blood pressure, 75/49 mmHg; heart rate, 122 beats per minute; and temperature, 39.1 °C. Her respiratory rate was 17 breaths per minute with 99 % oxygen saturation while breathing oxygen through a face mask at a rate of 10 litres per minute. Chest X-ray revealed a normal pattern. Transabdominal ultrasonography detected only a small amount of endometrial cavity fluid and no ascites. A speculum examination showed neither significant bleeding nor purulent discharge from the cervical os.

Laboratory tests revealed high levels of C-reactive protein (6.0 mg dl^−1^) and procalcitonin (20.45 ng ml^−1^). A white blood cell count of 4.4×10^9^ l^−1^, platelet count of 11×10^9^ l^−1^ and haemoglobin level of 90 g l^−1^ indicated thrombocytopenia with moderate anaemia. Although the prothrombin time and activated partial thromboplastin time were normal, fibrin/fibrinogen degradation products and elevated d-dimer levels were suggestive of pre-disseminated intravascular coagulation. Kidney and liver function results were normal. Blood, vaginal and sputum cultures were obtained, and piperacillin/tazobactam treatment was initiated (18 g day^−1^).

## Investigations

Gram-negative coccobacilli were detected in the blood by using a BacT/Alert blood culture system (Sysmex BioMérieux) and in the vaginal culture by inoculation on chocolate agar whereas sputum culture was negative. Isolates from blood and vaginal samples were identified as *H. influenzae* using a Vitek MS matrix-assisted laser desorption/ionization-time of flight MS system (Sysmex BioMérieux) and Haemophilus ID Quad Plate with Growth Factors (Becton Dickinson). These strains were verified as NTHi based on both slide agglutination with antisera (*H. influenzae* Antisera ‘SEIKEN’ Setl Denka Seiken) and PCR assays as previously described [11]. The biotype was determined as type III (ornithine decarboxylase- and indole-negative, urease-positive) using a rapid identification kit (ID Test HN-20 Rapid ‘Nissui’; Nissui Pharmaceutical). In addition, both strains tested negative for β-lactamase activity on the nitrocefin test (Showa Chemical). The MIC values for several antibiotics for the strain detected on blood culture were determined using the broth microdilution method in accordance with the guidelines published in the Clinical and Laboratory Standards Institute (CLSI) M07-A10 document [12]. MIC values were interpreted based on CLSI criteria M100-S27 [13] (Table 1). This strain was resistant to ampicillin. Alterations of penicillin-binding protein 3 (PBP3) were investigated by sequencing the region of the *ftsI* gene encoding the transpeptidase domain of PBP3, as previously described [14]. This strain showed amino acid substitutions in PBP3 (Asp350Asn, Ser357Asn, Met377Ile, Ser385Thr, Leu389Phe, Asn526Leu, Val562Leu and Asn569Ser) related to antimicrobial resistance. Together, these assays revealed BLNAR *H. influenzae* infection. The strains were also analysed by multilocus sequence typing (MLST) as previously described [15]. The allele and sequence type (ST) were assigned using the *H. influenzae* MLST website (http://haemophilus.mlst.net), which showed that the isolates from both blood and vaginal cultures belonged to ST425.

**Table 1.** MIC values for several antibiotics for the strain detected on blood culture The MICs were determined using the broth microdilution method in accordance with the guidelines published in the CLSI document M07-A10 [12]. MIC values were interpreted by CLSI criteria in strain M100-S27 [13]. R, resistant; S, susceptible; ABPC, ampicillin; ABPC/SBT, ampicillin/sulbactam;AMPC/CVA, amoxicillin/clavulate; CCL, cefaclor; CTX, cefotaxime; CDTR, cefditoren; LVFX, levofloxacin; CAM, clarithromycin.

	ABPC	ABPC/SBT	AMPC/CVA	CCL	CTX	LVFX	CAM
MIC value (µg ml^−1^)	8 R	8 R	>8 R	>16 R	2 S	≤0.5 S	8 S

## Treatment

Given the drug susceptibility of the blood culture strain and involvement of other anaerobic bacteria, piperacillin/tazobactam was discontinued and treatment with cefotaxime (6 g day^−1^) in combination with metronidazole (1.5 g day^−1^) was initiated. We performed anaerobic culture for the blood and vaginal samples, but no anaerobic bacteria were detected from these samples. However, anaerobes including of the *Bacteroides fragilis* group are known to be the causative pathogens for septic abortion and maternal sepsis [10, 16]. Cerotaxime has limited utility for some anaerobic bacteria, especially of the *Bacteroides fragilis* group [17]. We used metronidazole in combination with cefotaxime to treat anaerobic organisms. Intravenous infusion of noradrenaline (0.05 µg kg^−1^ min^−1^) was required to maintain an adequate blood pressure. Thrombomodulin (12800 U day^−1^) and antithrombin III (1500 U day^−1^) were also necessary as the patient developed disseminated intravascular coagulation during the course of treatment.

## Outcome and follow-up

The patient's condition gradually improved after several days of this treatment with careful monitoring, and she was ultimately discharged without sequelae or complaints 7 days after admission.

## Discussion

To our knowledge, this is the first case report of septic abortion caused by NTHi in Japan. Although a few cases of septic abortion due to *H. influenzae* have been previously published [18], causative serotypes have not been reported.

There are two possible *H. influenzae* transmission mechanisms that might lead to septic abortion. The first is haematogenous spread with subsequent infection of the placental amniotic fluid after primary maternal bacteraemia caused by invasion of this organism through the upper respiratory mucosa. An alternative possibility is ascending infection from the cervix and vagina. While *H. influenzae* usually colonizes human respiratory mucosal surfaces, this organism can also colonize the female reproductive tract where its isolation rate is typically 1–7 % or lower [19, 20]. In the case reported here, NTHi was detected in both blood and vaginal samples, but not in a sputum culture. Although the patient in this case showed symptoms of an upper respiratory tract infection initially, there was no chest X-ray findings of severe pneumonia that can cause bacteraemia. In the survey of invasive *H. influenzae* infections in Japan, most cases of bacteraemia in adults, but none in the age range 25–34 years, were due to pneumonia [21]. Thus, it seems less likely that *H. influenzae* invaded through the upper respiratory mucosa in this case. Therefore, we hypothesized that this infection arose from the ascending route. MLST, conducted to evaluate the clonal relationship between the blood and vaginal strains, revealed that both isolates belonged to ST425. Thus, these appear to be the same strain, demonstrating that ascending infection may be the likely route of transmission.

In severe viral maternal infection, such as influenza, dengue fever and human immunodeficiency virus, the maternal immune response to these viruses may result in spontaneous abortion without intrauterine infection [22]. On the other hand, there is little evidence that sepsis due to an acute bacterial infection is associated with placental abruption and spontaneous abortion. However, there remains the possibility that sepsis caused by *H. influenzae* infection could result in spontaneous abortion without intrauterine infection, since we could not assess conception nor amnion cultures. This patient entered septic shock immediately after abortion. The patient had light vaginal bleeding with lower abdominal pain for 4 days before abortion. It is less likely that the spontaneous abortion in this case was caused by overwhelming infection.

ST425 has only been found in a few adult cases of pneumonia in Spain [23] and a single case of newborn invasive disease in Italy [24]. Since genital tract infection by this ST is previously unknown, further investigation is required.

Biotyping classifies *H. influenzae* strains into seven biotypes, I–VII, according to biochemical test results [20]. The majority of Hib strains belong to biotype I, and NTHi mainly belongs to II or III [25]. Of these biotypes, NTHi biotype IV most commonly colonizes the genitourinary tract [19]. In addition, biotype IV strains are associated with genitourinary tract infections and neonatal sepsis [26, 27]. Recently, cases of meningitis and septicaemia in infants and toddlers caused by NTHi biotype III have been reported [28, 29]. Given the increasing prevalence of invasive NTHi disease, NTHi biotype III may become an important pathogen with the potential to cause such severe infections.

This is the first report of septic abortion caused by BLNAR *H. influenzae* globally. There are some case reports of septic abortions caused by NTHi [30, 31]. Although these reports did not mention β-lactamase productivities or antimicrobial susceptibility test results of *H. influenzae*, it is possible that BLNAR *H. influenzae* was included in cases of septic abortions. Because the MIC for cefotaxime of the strain isolated from blood was relatively high, we investigated alterations of PBP3 by sequencing the *ftsI* gene encoding the transpeptidase domain of PBP3. Eight amino acid substitutions in PBP3 between this strain and the *H. influenzae* Rd strain were found. This mutation pattern is one of the three major types found in BLNAR *H. influenzae* isolates from Japanese paediatric patients, and of the three, it causes the strongest antibiotic resistance [14, 32]. In Japan, BLNAR *H. influenzae* is widespread and has become a clinical concern, especially in paediatrics and otolaryngology [6]. Indeed, according to previous studies, more than half of *H. influenzae* isolates from paediatric patients are ampicillin-resistant, and most of those are BLNAR [7, 8]. The prevalence of BLNAR *H. influenzae* among adult women is not clear, but the case reported here suggests that it might be important to consider the possibility of BLNAR *H. influenzae* infection when pregnant or postpartum patients present with sepsis in Japan. Although penicillin with a β-lactamase inhibitor is currently recommended for the treatment of septic abortion [10], this combination will probably lead to treatment failure in the case of BLNAR *H. influenzae* infection.

This case reveals the importance of recognizing the pathogenicity of *H. influenzae* during pregnancy and the perinatal period. Although the number of reported invasive *H. influenzae* infections in Japanese women is low [5], this number might be underestimated due to the low sensitivity of the culture methods used. Since *H. influenzae* can cause septic abortion, these pathogens should be screened for and treated during pregnancy. Future investigation is warranted to determine the prevalence of genital colonization and the potential morbidity due to *H. influenzae* in pregnant women.
